# Cardiorenal Syndrome: Molecular Pathways Linking Cardiovascular Dysfunction and Chronic Kidney Disease Progression

**DOI:** 10.3390/ijms26157440

**Published:** 2025-08-01

**Authors:** Fabian Vasquez, Caterina Tiscornia, Enrique Lorca-Ponce, Valeria Aicardi, Sofia Vasquez

**Affiliations:** 1 Escuela de Nutrición y Dietética, Universidad Finis Terrae, Santiago 8320000, Chile; fvasquez@uft.cl; 2 Escuela de Enfermería, Universidad Finis Terrae, Santiago 8320000, Chile; elorca@uft.cl; 3 Escuela de Kinesiología, Facultad de Arte y Educación Física, Universidad Metropolitana en Ciencias de la Educación, Santiago 7760170, Chile; 4 Unidad de Diálisis, Clínica Indisa, Santiago 7501014, Chile; valeria.aicardi@gmail.com; 5 Escuela de Medicina, Universidad de Chile, Santiago 8380492, Chile; vasquezsofia36@gmail.com

**Keywords:** cardiorenal syndrome, chronic kidney disease, cardiovascular disease

## Abstract

Cardiorenal syndrome (CRS) is a multifactorial clinical condition characterized by the bidirectional deterioration of cardiac and renal function, driven by mechanisms such as renin–angiotensin–aldosterone system (RAAS) overactivation, systemic inflammation, oxidative stress, endothelial dysfunction, and fibrosis. The aim of this narrative review is to explore the key molecular pathways involved in CRS and to highlight emerging therapeutic approaches, with a special emphasis on nutritional interventions. We examined recent evidence on the contribution of mitochondrial dysfunction, uremic toxins, and immune activation to CRS progression and assessed the role of dietary and micronutrient factors. Results indicate that a high dietary intake of sodium, phosphorus additives, and processed foods is associated with volume overload, vascular damage, and inflammation, whereas deficiencies in potassium, magnesium, and vitamin D correlate with worse clinical outcomes. Anti-inflammatory and antioxidant bioactives, such as omega-3 PUFAs, curcumin, and anthocyanins from maqui, demonstrate potential to modulate key CRS mechanisms, including the nuclear factor kappa B (NF-κB) pathway and the NLRP3 inflammasome. Gene therapy approaches targeting endothelial nitric oxide synthase (eNOS) and transforming growth factor-beta (TGF-β) signaling are also discussed. An integrative approach combining pharmacological RAAS modulation with personalized medical nutrition therapy and anti-inflammatory nutrients may offer a promising strategy to prevent or delay CRS progression and improve patient outcomes.

## 1. Introduction

Cardiorenal syndrome (CRS) represents a complex and bidirectional pathological condition in which dysfunction in the heart or kidneys induces damage in the other organ, resulting in a vicious cycle that significantly increases morbidity and mortality [1,2].

Recently, it has been classified into five subtypes that reflect the directionality, chronicity, and simultaneity of the organ dysfunction between the heart and kidneys. Type 1 CRS involves acute cardiac dysfunction leading to acute kidney injury, while Type 2 refers to chronic heart failure resulting in chronic kidney disease (CKD). In contrast, Type 3 CRS begins with an acute kidney injury that triggers acute heart failure, and Type 4 describes the progressive impact of CKD on cardiac function. Finally, Type 5 CRS occurs when both organs are simultaneously affected by an acute or chronic systemic condition, such as sepsis or autoimmune diseases [3].

The intricate interplay between cardiovascular disease (CVD) and CKD is underpinned by shared pathophysiological mechanisms, including the sustained activation of the renin–angiotensin–aldosterone system (RAAS), systemic inflammation, oxidative stress, endothelial dysfunction, mitochondrial impairment, and progressive tissue fibrosis [4,5,6].

The chronic stimulation of the RAAS not only elevates blood pressure and promotes sodium retention but also contributes to myocardial hypertrophy, vascular remodeling, and glomerular injury. Angiotensin II and aldosterone play central roles in promoting pro-inflammatory, pro-oxidative, and profibrotic cascades, exacerbating multiorgan dysfunction [7]. Oxidative stress further amplifies endothelial damage through the excessive generation of reactive oxygen species (ROS) and the reduction in nitric oxide (NO) bioavailability, compromising vasodilation and accelerating atherosclerosis [8,9]. These effects are worsened in CKD, where mitochondrial dysfunction and the accumulation of uremic toxins such as indoxyl sulfate and p-cresyl sulfate exacerbate ROS production and vascular inflammation [10,11].

Systemic inflammation, mediated by cytokines like interleukin-6 (IL-6), tumor necrosis factor-alpha (TNF-α), and the activation of the NLRP3 inflammasome, plays a pivotal role in the progression of CRS. This inflammatory milieu promotes the release of profibrotic factors, including transforming growth factor-beta (TGF-β), driving cardiac and renal tissue remodeling [12,13].

Furthermore, aging, hypertension (HTN), and diabetes mellitus (DM), particularly diabetic kidney disease, contribute to the susceptibility and progression of CRS. These conditions amplify metabolic dysregulation, endothelial dysfunction, and immune activation, accelerating multiorgan deterioration [14,15].

Emerging evidence suggests that targeted interventions addressing these mechanisms, including RAAS inhibition, antioxidant and anti-inflammatory therapies, gene-based strategies, and individualized nutritional interventions, may play a crucial role in attenuating the progression of CRS and improving clinical outcomes [16,17].

This review explores the key molecular mechanisms involved in the pathogenesis of CRS, the influence of genetic and metabolic risk factors, and the potential of integrative therapeutic approaches, with a particular emphasis on the role of nutritional strategies to mitigate systemic inflammation, oxidative stress, and organ fibrosis.

## 2. Materials and Methods

This narrative review was structured following a systematic approach to ensure methodological rigor and transparency throughout the process. A comprehensive literature search was conducted between February and May 2025 using the electronic databases PubMed, Scopus, ScienceDirect, Google Scholar, and Medline and was complemented by consultation of university libraries. The following MeSH terms and Boolean combinations were used: “cardiorenal syndrome” AND “heart failure” OR “chronic kidney disease” OR “renal dysfunction” OR “cardiovascular disease” AND “inflammation” OR “oxidative stress” OR “fibrosis” AND “pathophysiology” OR “biomarkers” AND “treatment” OR “RAAS blockade” OR “SGLT2 inhibitors” OR “inflammatory mediators”.

To identify relevant literature, the reference lists and “cited by” sections of selected studies were also reviewed. The inclusion criteria were as follows: (1) studies published up to April 2025; (2) original articles, systematic reviews, and meta-analyses conducted in humans or relevant animal models focused on cardiorenal syndrome; (3) publications addressing pathophysiological mechanisms, biological markers, or therapeutic interventions in the cardiorenal context; and (4) studies published in English or Spanish. Articles without full-text access, those irrelevant to the topic, duplicates, and non-indexed publications were excluded.

The selection process consisted of an initial removal of duplicates, followed by title and abstract screening. Subsequently, full-text articles were assessed to confirm compliance with the inclusion criteria. Data extraction was conducted in a standardized manner, including the following: study type, year of publication, sample size, population characteristics, type and duration of the intervention (if applicable), evaluated biomarkers (inflammation, oxidative stress, fibrosis, endothelial dysfunction), and primary outcomes related to cardiac, renal, or combined function. Reported adverse effects and dose–response relationships were also recorded.

Findings were organized into thematic categories (pathophysiology, diagnostic/prognostic biomarkers, and emerging therapies) and grouped by study type (preclinical vs. clinical), followed by a critical narrative synthesis based on methodological robustness and clinical relevance. Particular emphasis was placed on high-impact studies published between 2021 and 2025 that explored the interplay among systemic inflammation, mitochondrial dysfunction, neurohormonal activation, and cardiac or renal remodeling.

Discrepancies among authors regarding study selection or quality assessment were resolved through consensus discussions, thereby enhancing the consistency and validity of the review. Finally, technical language was carefully selected to support multidisciplinary understanding, prioritizing conceptual precision and avoiding unnecessary redundancy.

## 3. Key Molecular Mechanisms in the Interrelation Between Cardiovascular Disease and Chronic Kidney Disease

### 3.1. Role Renin–Angiotensin–Aldosterone System in CVD and CKD

The RAAS is a critical hormonal pathway involved in the regulation of blood pressure, extracellular fluid volume, and electrolyte homeostasis. It plays a pivotal role in the pathophysiology of CRS, a condition characterized by the pathological interplay between CVD and CKD [1]. The classical RAAS cascade is initiated by the renin release from the juxtaglomerular apparatus in response to reduced renal perfusion or cardiac output. Renin converts hepatic angiotensinogen to angiotensin I, which is subsequently cleaved by the angiotensin-converting enzyme (ACE), primarily in the pulmonary endothelium, into angiotensin II (Ang II), a potent vasoconstrictor that binds to angiotensin type 1 receptors (AT1Rs), elevating systemic blood pressure and stimulating aldosterone secretion from the adrenal cortex [18,19,20].

Ang II not only induces vasoconstriction and increases the afterload but also promotes vascular smooth muscle cell proliferation, arterial stiffness, and atherosclerosis [21,22], while concurrently stimulating the production of pro-inflammatory cytokines (e.g., TNF-α, IL-6) and reactive oxygen species (ROS), ultimately impairing endothelial function [23]. Aldosterone, in turn, contributes to sodium retention and potassium excretion, exacerbating hypertension, fluid overload, and systemic congestion, key features of both CVD and CKD [24,25]. Beyond its classic renal effects, aldosterone also exerts genomic actions by regulating the expression of sodium transporters and non-genomic effects through rapid intracellular signaling pathways that trigger inflammation and fibrosis [26,27].

Importantly, a nonclassical RAAS axis has also been described, involving the ACE2-mediated conversion of Ang II into angiotensin-(1–7) [Ang(1–7)], which binds to the Mas receptor, exerting vasodilatory, antifibrotic, and anti-inflammatory effects. This counter-regulatory arm opposes the deleterious actions of the ACE/Ang II/AT1R axis and is considered protective in the context of cardiovascular and renal disease [28,29,30].

In the context of CRS, chronic RAAS activation precipitates myocardial fibrosis, left ventricular hypertrophy, and progressive renal damage. Ang II and aldosterone upregulate profibrotic mediators such as the transforming growth factor-beta (TGF-β) and connective tissue growth factor (CTGF), promoting extracellular matrix accumulation and organ fibrosis [31]. In the kidney, these effects contribute to glomerulosclerosis, podocyte injury, mesangial proliferation, and albuminuria hallmarks of CKD progression [32,33,34].

Even in the absence of overt HTN, an aldosterone excess can induce myocardial remodeling and renal injury, highlighting its pathogenic role in target organ damage [23].

Altogether, RAAS overactivation represents a convergent mechanism linking cardiovascular and renal dysfunction, with Ang II and aldosterone serving as central mediators of inflammation, fibrosis, and hemodynamic stress. Therapeutic strategies aimed at inhibiting RAAS components or enhancing the ACE2/Ang(1–7)/Mas receptor axis offer promising avenues for mitigating the progression of CRS and improving patient outcomes.

### 3.2. Oxidative Stress and Endothelial Dysfunction

Oxidative stress and endothelial dysfunction are interrelated processes that play a critical role in the development and progression of CRD. The endothelium, a monolayer of cells lining the inner surface of blood vessels, regulates key vascular functions including vasodilation, coagulation, and inflammatory responses.

Endothelial dysfunction, characterized by reduced nitric oxide (NO) bioavailability, serves as an early marker of the vascular pathology and is strongly associated with an increased cardiovascular risk [8,35]. Oxidative stress, defined as an imbalance between the production of reactive oxygen species (ROS) and antioxidant defenses, induces structural damage to lipids, proteins, and DNA and is a central mechanism in the pathogenesis of atherosclerosis [9,36].

In CKD, mitochondrial dysfunction impairs electron transport chain activity, leading to excessive ROS generation [10,36]. These elevated ROS levels deplete antioxidant reserves, such as reduced glutathione, and damage key cellular components, a process further exacerbated by a diminished systemic antioxidant capacity in CKD patients [37]. ROS also react with NO to form peroxynitrite, a highly reactive oxidant that further reduces NO availability, thereby impairing endothelium-dependent vasodilation, promoting vasoconstriction and hypertension, and advancing vascular injury [38,39,40].

Metabolic factors such as hyperglycemia and insulin resistance have also been shown to enhance ROS production and suppress NO synthesis, aggravating endothelial damage and accelerating atherosclerotic processes [41]. Chronic inflammation compounds these effects, as pro-inflammatory cytokines such as IL-6 and TNF-α stimulate ROS production and reduce NO availability, creating a self-reinforcing cycle of endothelial dysfunction [42]. In addition, uremic toxins including indoxyl sulfate and p-cresyl sulfate, which accumulate in CKD, have been shown to increase ROS generation in endothelial cells, activate the RhoA/ROCK signaling pathway, and promote endothelial senescence and inflammation [10,11,42,43]. These toxins also suppress the expression of antioxidant enzymes such as superoxide dismutase (SOD) and activate pro-inflammatory pathways including nuclear factor-κB (NF-κB), thus amplifying oxidative injury and endothelial dysfunction [44,45].

Moreover, aging is associated with increased oxidative stress and decreased endothelial function even in the absence of overt cardiovascular disease. This decline contributes to an impaired vasodilatory capacity and an increased vulnerability to external insults, partially explaining the higher incidence of cardiovascular events among older adults [46].

Hyperhomocysteinemia, characterized by elevated plasma homocysteine levels, has also been identified as an independent cardiovascular risk factor. Homocysteine induces oxidative stress and endothelial injury, thereby contributing to endothelial dysfunction and the progression of atherosclerosis [47].

### 3.3. Inflammatory Mechanisms Involved Between CVD and CKD

Monocytes and macrophages are key players in the inflammatory response, and their dysregulation is a hallmark of CKD-associated inflammation. In CKD, monocytes exhibit increased oxidative stress and pro-inflammatory cytokine production, which contributes to the development of atherosclerosis [48]. Specifically, intermediate monocytes (IMs) with a high HLA-DR (Human Leukocyte Antigen—DR isotype) expression are expanded in CKD patients and exhibit enhanced adhesion to endothelial cells, promoting vascular inflammation [49].

Increased levels of ROS in CKD patients lead to the activation of pro-inflammatory pathways, including the NF-κB pathway, which upregulates the production of pro-inflammatory cytokines such as TNF-α and IL-6 [50]. Furthermore, oxidative stress promotes the formation of advanced oxidation protein products (AOPPs), which are potent stimulators of inflammatory responses in monocytes and macrophages [51].

The NLRP3 inflammasome, a multiprotein complex that activates pro-inflammatory cytokines such as IL-1β and IL-18, has been the focus of increasing attention since early studies in the 2010s due to its role in sterile inflammation in CKD and CVD [52,53,54,55]. The NLRP3 inflammasome has been identified as a pivotal regulator in both inflammation and oxidative stress across a variety of cardiorenal disease contexts, influencing disease progression through diverse but interrelated mechanisms [53].

The extant literature suggests that the activation of the NLRP3 inflammasome significantly increases the production of pro-inflammatory cytokines, such as IL-1β and IL-18, in cardiac and renal cells, thereby contributing to the progression of the disease [56,57]. Similarly, NLRP3 inflammasome activation has been demonstrated to be closely linked with increased reactive ROS production and impaired antioxidant responses, driving oxidative damage in cardiac and renal tissues [53,58]. Mitochondrial dysfunction and ROS generation were frequently reported as effects of NLRP3 inflammasome signaling [59].

Antioxidant pathways involving Nrf2 have been demonstrated to modulate NLRP3 activation and reduce oxidative stress, thus indicating potential therapeutic targets [53,60].

### 3.4. Overview of Mitochondrial Dysfunction in CKD and CVD

The kidney is characterized by an abundance of mitochondria, which are essential for the maintenance of high energy demands, particularly in proximal tubular cells that are responsible for reabsorption and secretion processes. The mitochondria within these cells are responsible for generating most of the ATP necessary for solute transport, thereby maintaining cellular homeostasis. Any disruption in mitochondrial function has the potential to impair renal function, consequently leading to CKD [61,62].

The following characteristics are indicative of mitochondrial dysfunction in cases of CKD:

1. Impaired Bioenergetics: There has been a reduction in oxidative phosphorylation (OXPHOS) efficiency and a decrease in ATP production. This is typically due to defects in the mitochondrial respiratory chain and the tricarboxylic acid (TCA) cycle [63,64].
2. Oxidative Stress: The overproduction of ROS and the subsequent diminution of antioxidant defenses result in mitochondrial damage. ROS has the potential to cause damage to mitochondrial DNA, proteins, and lipids, which can further exacerbate dysfunction [44,65].
3. Mitochondrial Dynamics: Imbalances in the processes of mitochondrial fission and fusion have been demonstrated to result in mitochondrial fragmentation. This phenomenon has been observed to be associated with the process of apoptosis and the progression of disease [66,67].
4. Mitophagy and Biogenesis: Impaired mitophagy (the removal of damaged mitochondria) and reduced mitochondrial biogenesis (the generation of new mitochondria) are pivotal factors in the development of mitochondrial dysfunction in cases of CKD [61,68].

As previously mentioned, the consequences of mitochondrial dysfunction in CKD include energy repletion, where the reduced ATP production impairs tubular reabsorption and promotes tubulointerstitial fibrosis, a hallmark of CKD progression [62,64]. Additionally, oxidative stress and inflammation have been demonstrated to play a pivotal role in the progression of renal diseases. Increased ROS levels have been shown to trigger inflammatory pathways, resulting in further damage to renal tissue and contributing to disease progression [44,65]. Also, muscle wasting and metabolic acidosis have been identified as significant contributors to the severity of the condition. Sarcopenia and metabolic acidosis, secondary to CKD, have been found to lead to impaired physical function and overall health [69].

Furthermore, the heart is another organ with high energy demands, relying almost exclusively on mitochondrial ATP production to maintain contractile function. Mitochondrial dysfunction in the heart has been proven to be a significant contributing factor to CVD, encompassing conditions such as heart failure and cardiomyopathy [70,71].

The underlying causes of mitochondrial dysfunction in CVD are as follows:

1. Impaired Oxidative Phosphorylation: The reduced efficiency of the mitochondrial respiratory chain and decreased ATP production have been demonstrated to impair cardiac contractility and promote cell death [71,72].
2. Lipotoxicity and Metabolic Stress: The accumulation of toxic lipids and imbalances in cellular energy metabolism create a state of mitochondrial dysfunction that exacerbates oxidative stress, promotes inflammation, and contributes to progressive myocardial damage, negatively impacting cell viability and contractile function [71,72,73,74].
3. Mitochondrial Dynamics and Biogenesis: The process of mitochondrial fragmentation and dysfunction in the heart is the result of increased mitochondrial fission and reduced biogenesis [66,70].
4. Oxidative Stress and Inflammation: As is the case of CKD, oxidative stress is central to the etiology of mitochondrial dysfunction, which in turn promotes inflammation and tissue damage in the cardiovascular system [44,72].

Consequently, the consequences of mitochondrial dysfunction in CVD include cardiac energy depletion, where the reduced ATP production impairs cardiac contractility, leading to heart failure and arrhythmias [71,73] and vascular dysfunction, where mitochondrial dysfunction in endothelial cells contributes to impaired vasodilation and increased vascular resistance, exacerbating hypertension and atherosclerosis [70,71].

Concurrently, mitochondrial dysfunction in CKD and CVD results in diminished ATP production, which in turn impairs cellular function and promotes tissue damage. In the kidneys, this results in impaired solute reabsorption and tubular injury, while in the heart, it leads to reduced contractility and cardiac failure [61,73]. Moreover, mitochondrial dysfunction contributes to metabolic acidosis, which in turn impairs insulin signaling and promotes insulin resistance. This process has been shown to induce a catabolic state, which in turn accelerates muscle wasting and the progression of the disease [62,69]. In a similar manner, the accumulation of lipids in mitochondrial membranes disrupts the energy metabolism and increases ROS production, which in turn causes further damage to the mitochondria and perpetuates dysfunction. This process is particularly relevant in both CKD and CVD [71,74].

The bidirectional relationship between CKD and CVD is often referred to as the CRS. Mitochondrial dysfunction plays a crucial role in this interplay, as both organs are highly dependent on mitochondrial energy production. Dysfunction in one organ has been demonstrated to exacerbate disease in another, thereby creating a vicious cycle of progression [70,75].

Finally, it is important to note that there is a link between these two pathologies, with both CKD and CVD being characterized by increased oxidative stress and inflammation.

This is driven by mitochondrial dysfunction [44,65]. Furthermore, metabolic reprogramming is a key factor in the progression of both CKD and CVD. This term refers to alterations in the mitochondrial metabolism, including impaired fatty acid oxidation and increased glycolysis [71,76].

## 4. Risk Factors and Genetic Predisposition to Cardiorenal Syndrome

**Figure 1:**
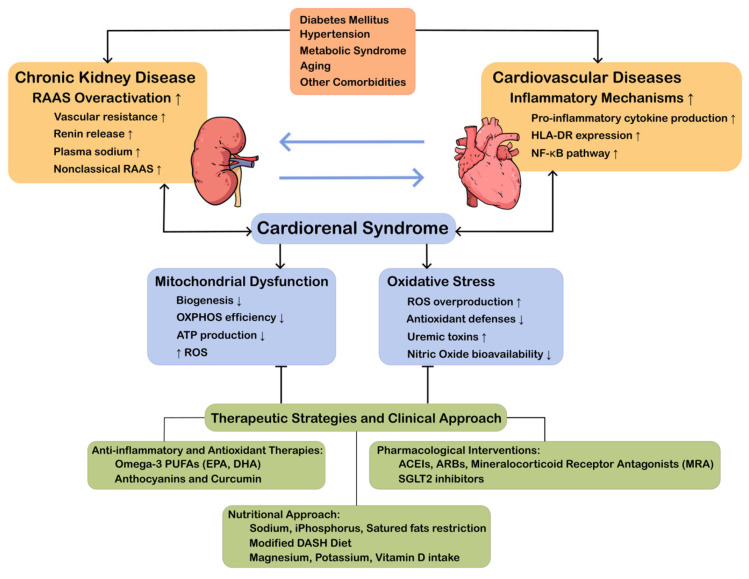
Cardiorenal Syndrome: The mind map provides a visual representation of the pathophysiological mechanisms involved, including RAAS overactivation, oxidative stress, and inflammation. It emphasizes the bidirectional heart–kidney connections and offers a comprehensive range of therapeutic strategies, including pharmacological modulation, nutritional interventions, and emerging therapies, to enhance patient outcomes. RAAS: Renin–Angiotensin–Aldosterone System; HLA-DR: Human Leukocyte Antigen; NF-κB: Nuclear Factor-Kappa B; OXPHOS: Oxidative Phosphorylation; ROS: Reactive Oxygen Species; PUFAs: Polyunsaturated Fatty Acids; EPA: Eicosapentaenoic Acid; DHA: Docosahexaenoic Acid; ACEIs: Angiotensin-Converting Enzyme Inhibitors; ARBs: Angiotensin II Receptor Blockers; and DASH: Dietary Approaches to Stop Hypertension.

CRS arises from a complex interaction between molecular mechanisms and clinical risk factors, where genetic, metabolic, and environmental elements converge to facilitate its onset and progression. Identifying these risk factors is essential for implementing effective preventive, therapeutic, and prognostic strategies in vulnerable populations. The above is illustrated in Figure 1.

### 4.1. The Influence of Family History and Genetics on the Development of Cardiorenal Syndrome

Genetic predisposition plays a significant role in the susceptibility to CRS, particularly in individuals with a family history of CVD, CKD, hypertension, or type 2 diabetes mellitus. Multiple genetic variants have been associated with an increased risk of cardiac or renal dysfunction by altering key regulatory pathways such as the RAAS, oxidative stress, inflammation, and fibrosis.

For instance, mutations in the *PKD1* gene, responsible for ADPKD, are associated with a higher risk of left ventricular hypertrophy, diastolic dysfunction, and cardiovascular events [76]. Similarly, polymorphisms in genes such as *ACE*, *AGT*, *NOS3*, and *TGF-β1* have been shown to modulate the expression of key mediators in myocardial and glomerular fibrotic progression [77,78,79,80]. These findings suggest that family history is not merely an epidemiological marker, but also an opportunity for early monitoring and precision prevention strategies.

### 4.2. Diabetes Mellitus and Hypertension as Key Risk Factors

DM and HTN are the two most widely recognized risk factors in the pathogenesis of CRS, due to their high global prevalence and their direct impact on the cardiac and renal structure and function. Chronic hyperglycemia in DM promotes the formation of advanced glycation end-products (AGEs), endothelial dysfunction, systemic inflammation, and structural damage to both glomeruli and vasculature [41]. In parallel, HTN induces left ventricular hypertrophy, glomerular sclerosis, and sustained RAAS activation, creating a pathophysiological environment conducive to cross-organ dysfunction.

Longitudinal studies have shown that the coexistence of DM and HTN not only accelerates the progression to chronic kidney failure and heart failure but also increases the cardiovascular mortality risk in patients with advanced CKD [14,81]. Moreover, these conditions are associated with metabolic disturbances that exacerbate oxidative stress, insulin resistance, mitochondrial dysfunction, and vascular remodeling, reinforcing their role as central etiological drivers of CRS.

### 4.3. Impact of Aging and Comorbidities on Cardiorenal Syndrome

Aging is a non-modifiable determinant of CRS that affects both cardiac and renal function, even in the absence of overt disease. Aging is associated with a progressive decline in the glomerular filtration rate, increased arterial stiffness, loss of functional myocardial mass, and reduced endothelial vasodilatory and antioxidant capacity [46]. These physiological changes increase the vulnerability to external insults and the accumulation of subclinical injuries, facilitating the transition to overt organ dysfunction.

Additionally, the presence of comorbidities such as obesity, metabolic syndrome, dyslipidemia, chronic obstructive pulmonary disease (COPD), and frailty amplifies systemic inflammation, hemodynamic stress, and neurohormonal activation, creating a clinical scenario in which CRS progression is more rapid and severe [15]. The accumulation of these risk factors in older adults underscores the need for comprehensive prevention strategies, early diagnosis, and multidisciplinary treatment approaches.

## 5. Therapeutic Strategies for the Management of Cardiorenal Syndrome

The management of CRS requires a comprehensive therapeutic approach that integrates pharmacological interventions, nutritional therapies, anti-inflammatory strategies, and, in selected cases, emerging modalities such as gene therapy. Given the complex pathophysiology of this condition, which encompasses the activation of the RAAS, oxidative stress, chronic inflammation, mitochondrial dysfunction, and progressive fibrosis, its management must be multifactorial and personalized.

### 5.1. The Modulation of the RAAS and Antihypertensive Therapies

The pharmacological inhibition of the RAAS is a well-established strategy to slow the progression of both cardiovascular and renal disease. Angiotensin-converting enzyme inhibitors (ACEIs), angiotensin II receptor blockers (ARBs), and mineralocorticoid receptor antagonists have been shown to reduce blood pressure, proteinuria, and myocardial and glomerular remodeling [4,81,82].

The introduction of sodium–glucose co-transporter 2 inhibitors (SGLT2is), such as dapagliflozin and empagliflozin, has demonstrated benefits beyond glycemic control. These include reductions in the blood pressure, plasma volume, and RAAS activation, along with improvements in renal and cardiac function—even in patients without diabetes [83].

Given that hypertension is both a cause and a consequence of CRS, it is critical to control the sodium intake (<2.3 g/day) and maintain an adequate balance of potassium, calcium, and magnesium. A modified DASH (Dietary Approaches to Stop Hypertension) diet rich in fruits, vegetables, and low-fat dairy has shown significant antihypertensive effects in patients with CKD and heart failure, though it must be carefully adapted to the renal functional status [84].

### 5.2. Anti-Inflammatory and Antioxidant Therapies

Systemic inflammation and oxidative stress are central drivers of endothelial dysfunction, progressive fibrosis, and multiorgan damage in CRS. Nutritional deficits in dietary antioxidants, such as vitamins C and E, and polyphenols, combined with a high intake of saturated fats and ultra-processed foods, have been shown to enhance the production of AGEs and ROS, exacerbating both renal and cardiovascular injury [17,85].

Several bioactive compounds, including omega-3 polyunsaturated fatty acids (PUFAs), flavonoids, and curcumin, have demonstrated anti-inflammatory and antioxidant properties, as well as potential modulatory effects on the RAAS, suggesting their therapeutic potential in the inflammatory–oxidative context of CRS [17].

Among these, omega-3 PUFAs, primarily eicosapentaenoic acid (EPA) and docosahexaenoic acid (DHA), have gained particular interest. These fatty acids exert powerful anti-inflammatory, antioxidant, and cardioprotective effects, which are highly relevant in CRS, a condition characterized by chronic low-grade inflammation and endothelial dysfunction. Multiple randomized clinical trials and observational studies have shown that an omega-3 supplementation significantly reduces inflammatory biomarkers such as C-reactive protein (CRP), IL-6, and TNF-α in patients with CKD and heart failure [86,87,88].

EPA and DHA have also been shown to inhibit NLRP3 inflammasome activation in human adipose tissue, reduce IL-1β secretion, and enhance the biosynthesis of specialized pro-resolving lipid mediators (SPMs), including resolvins and protectins, key drivers in resolving chronic inflammation [86,89]. In hemodialysis patients, an omega-3 supplementation (up to 4 g/day) improved lipid profiles and reduced vascular inflammation markers [87,90]. The ORENTRA trial further supported these findings, demonstrating reduced cellular senescence markers in renal transplant recipients receiving omega-3s, highlighting their cytoprotective and anti-aging potential in high-risk renal populations [88,91].

Although some heterogeneity exists in dosing and intervention durations, the cumulative evidence strongly supports the role of omega-3s in modulating immune responses, improving endothelial function, and potentially slowing the progression of CRS. These findings advocate for the inclusion of omega-3s, whether through dietary intake (e.g., oily fish) or oral supplementation, as a valuable nutritional strategy in CRS management.

Another promising group of compounds are anthocyanins, flavonoids found in berries such as maqui (*Aristotelia chilensis*). These polyphenols have shown a potent antioxidant and anti-inflammatory activity by suppressing the NF-κB and COX-2 expression, reducing lipid peroxidation, and improving adipokine and glycemic profiles [92]. In animal models of CKD, a maqui supplementation reduced glomerular fibrosis, improved lipid profiles, lowered pro-inflammatory cytokine levels, and preserved renal function. Key anthocyanins such as cyanidin-3-O-glucoside (C3G) and delphinidins were shown to inhibit lipid-induced NF-κB activation and mitigate oxidative injury in renal tubular cells [93].

In human studies, maqui extract (60–180 mg/day) improved insulin sensitivity, fasting glucose, and oxidative stress markers such as 8-iso-PGF2α, suggesting potential benefits in the early stages of CRS, before the establishment of irreversible structural damage [94].

In addition to berry-derived polyphenols, curcumin, the main bioactive compound in *Curcuma longa*, has shown relevant antioxidant and anti-inflammatory effects in chronic diseases. Curcumin modulates key inflammatory pathways, including the inhibition of NF-κB activation, reduction in ROS, and downregulation of cytokines such as TNF-α, IL-1β, and IL-6 [95]. It also improves endothelial function by increasing NO bioavailability, reducing mitochondrial dysfunction, and attenuating renal damage in animal models of CKD [96,97]. These findings position curcumin as a potential adjunct in CRS therapy, particularly within antioxidant and anti-inflammatory dietary strategies.

Collectively, anti-inflammatory and antioxidant therapies, especially those based on natural compounds such as omega-3s, anthocyanins, and curcumin, offer promising adjunctive options in CRS management. However, further clinical trials are necessary to define optimal dosages, long-term safety, and synergistic effects with conventional treatments.

### 5.3. Gene Therapies

Current gene therapy approaches for treating CRS are still in the exploratory phase, with a focus on addressing the complex interplay between cardiac and renal dysfunction. These therapies target the underlying pathophysiological mechanisms that exacerbate CRS, such as neurohormonal activation and oxidative stress [98,99].

There are two main types of gene therapy approaches for CRS: vector-based and RNA-directed strategies [100,101]. The use of antisense oligonucleotides that target microRNA-21 through RNA interference is suggested by the evidence [100], while others employ adeno-associated virus vectors to deliver genes, most notably those encoding sarcoplasmic/endoplasmic reticulum calcium ATPase 2a, as well as small interfering RNAs [100,102,103].

Modern genetic interventions focus on modulating the pathways linking heart and kidney problems. Adeno-associated viruses (AAVs) are used a lot as vectors because they are safe and can be used to express genes in the heart for a long time [102]. The core strategy for treating heart disease involves promoting new blood vessel growth using factors like the vascular endothelial growth factor (VEGF) and fibroblast growth factor (FGF) [102,104,105]. Another significant area is enhancing myocyte calcium handling and cardiac contractility through the overexpression of proteins like SERCA2a, S100A1, and adenylyl cyclase type 6 (AC6) [102]. Finally, several significant biomarkers have been identified as the direct or indirect target of these gene therapies. Among these biomarkers, microRNA-21 has been selected for investigation as a potential therapeutic target for the reduction in cardiac and renal fibrosis [100,103,104,105].

### 5.4. Nutritional Approach

Nutrition plays a central role in both the prevention and management of CRS. An excessive dietary intake of sodium; inorganic phosphorus, commonly present as additives in processed meats (such as deli ham, sausages, bacon, and hot dogs) and carbonated beverages (like colas and flavored sodas); saturated fats (found in full-fat dairy products, butter, lard, and red meats); and refined sugars (present in candy, pastries, sweetened cereals, commercial baked goods, and sugar-sweetened beverages) has been closely linked to systemic inflammation, volume overload, vascular calcification, and endothelial dysfunction [106,107].

These dietary patterns also impair NO bioavailability and worsen oxidative stress. Simultaneously, diets low in fruits, vegetables, fiber, and antioxidant-rich compounds reduce the body’s capacity to counteract oxidative damage [17,108].

Among these factors, the sodium intake plays a particularly critical role in the CRS pathophysiology. A high sodium consumption contributes directly to extracellular fluid expansion, HTN, left ventricular hypertrophy, and increased albuminuria, key features in both CKD and heart failure. Excess sodium has also been shown to potentiate oxidative stress, increase sympathetic nervous system activity, and enhance RAAS activation, thereby amplifying cardiovascular and renal injury. Evidence suggests that sodium restriction (<2.3 g/day) not only lowers blood pressure but also improves arterial compliance, reduces proteinuria, and enhances the efficacy of antihypertensive medications [17,108]. Moreover, a dietary sodium excess has been associated with impaired immune regulation and pro-inflammatory cytokine release, further aggravating systemic inflammation in CRS.

From a micronutrient perspective, deficiencies in magnesium, potassium, and vitamin D are commonly observed in patients with CRS and are associated with poorer clinical outcomes.

Magnesium exerts key vasodilatory effects in the context of cardiorenal syndrome by acting as a calcium antagonist in vascular smooth muscle, stimulating nitric oxide production via eNOS activation, inhibiting the release of angiotensin II and catecholamines, and reducing endothelial inflammation and oxidative stress. These mechanisms help improve endothelial function and lower peripheral vascular resistance [108].

In patients with chronic kidney disease and those undergoing hemodialysis, magnesium deficiency is common and is associated with an increased arterial stiffness, endothelial dysfunction, and cardiovascular risk, underscoring the importance of adequate monitoring and supplementation [109].

Potassium, often restricted in advanced stages of CKD, plays a fundamental role in blood pressure regulation and endothelial function. When appropriately reintroduced based on renal function and serum levels, the potassium intake may enhance blood pressure control and reduce vascular stiffness [110]. Vitamin D, in addition to its essential function in the mineral metabolism, also modulates RAAS activity and exerts anti-inflammatory effects; its deficiency is highly prevalent in patients with diabetes and CKD, especially those with diabetic nephropathy [111].

In patients with diabetic kidney disease, specific nutritional strategies should include achieving glycemic control through low-glycemic index carbohydrates, a moderate protein intake (0.6–0.8 g/kg/day) with an emphasis on plant-based sources, and the careful monitoring of zinc, chromium, and vitamin B12 levels—especially in individuals using metformin [112,113].

Finally, personalized MNT must be integrated into the comprehensive management of CRS. MNT should be dynamically adjusted to the patient’s renal stage, metabolic status, and cardiovascular risk profile. Tools such as the SGA, the MIS, and biochemical markers for micronutrient deficiencies and PEW are essential for nutritional monitoring and decision-making [114].

## 6. Discussion

CRS epitomizes a multifactorial and progressive disorder in which cardiac and renal dysfunctions are interdependently amplified, leading to high morbidity and mortality rates. Central to this interrelationship is the chronic activation of the RAAS, which contributes to hemodynamic instability, vascular remodeling, sodium retention, and fibrosis in both cardiac and renal tissues [1,18,20].

The upregulation of angiotensin II and aldosterone further exacerbates inflammation and oxidative stress, thereby perpetuating endothelial dysfunction and promoting organ damage [23].

Oxidative stress and endothelial dysfunction emerge as key mediators in CRS. Excess ROS generation, particularly in CKD patients due to mitochondrial dysfunction, impairs NO bioavailability and activates pro-inflammatory signaling such as NF-κB, fueling a self-sustaining loop of vascular injury [36,37,38]. Uremic toxins like indoxyl sulfate and p-cresyl sulfate further enhance ROS production and trigger endothelial senescence via RhoA/ROCK pathways [10,11].

Moreover, inflammatory pathways play a prominent role. Monocyte activation, IL-6, TNF-α production, and NLRP3 inflammasome activation are heightened in CRS, contributing to vascular and myocardial fibrosis [12,13,52]. Age-related oxidative stress and immune dysfunction compound these alterations, increasing susceptibility in elderly populations [46].

Recent studies highlight the relevance of nutritional interventions in modulating these pathophysiological pathways. Omega-3 polyunsaturated fatty acids (PUFAs), particularly EPA and DHA, have shown anti-inflammatory and antioxidant effects by reducing IL-6, TNF-α, and CRP levels and by inhibiting NLRP3 inflammasome activation, especially in CKD and dialysis patients [86,90]. Likewise, flavonoids such as anthocyanins and curcumin attenuate oxidative stress, enhance NO bioavailability, and suppress pro-inflammatory gene expression via NF-κB inhibition [93,95].

Dietary patterns rich in sodium, phosphorus additives, and processed foods have been associated with vascular calcification, volume overload, and systemic inflammation, while deficiencies in potassium, magnesium, and vitamin D correlate with worse outcomes in CKD and CVD populations [17,108,109].

Finally, although still experimental, gene therapy targeting eNOS expression or TGF-β inhibition has shown promising antifibrotic effects in preclinical models, offering a future avenue for personalized medicine in CRS [98,104].

## 7. Conclusions

CRS represents a complex and multifactorial clinical condition characterized by the interplay between cardiac and renal dysfunction. Its progression is driven by key pathophysiological mechanisms such as the overactivation of the RAAS, oxidative stress, chronic inflammation, endothelial dysfunction, and fibrosis. Advances in molecular understandings have highlighted the central role of immune and metabolic dysregulation in this bidirectional organ failure. Nutritional interventions, particularly those involving omega-3 fatty acids, anthocyanin-rich extracts like *Aristotelia chilensis*, and curcumin, have shown potential in modulating key inflammatory and oxidative pathways, including NF-κB and the NLRP3 inflammasome. Moreover, correcting micronutrient deficiencies and limiting dietary components such as sodium and phosphorus additives may help attenuate the volume overload, vascular injury, and systemic inflammation. While pharmacological RAAS modulation remains foundational, emerging strategies such as gene therapy and personalized MNT may offer synergistic benefits. A comprehensive, integrative, and individualized approach targeting both systemic and nutritional drivers is essential to improving outcomes and the quality of life in patients with CRS.

## Abbreviations

The following abbreviations are used in this manuscript:

AC6	Adenylyl Cyclase Type 6
ACE	Angiotensin-Converting Enzyme
ACEIs	Angiotensin-Converting Enzyme Inhibitors
ADPKD	Autosomal Dominant Polycystic Kidney Disease
AGEs	Advanced Glycation End-Products
Ang II	Angiotensin II
Ang(1–7)	Angiotensin-(1–7)
ARBs	Angiotensin II Receptor Blockers
AT1R	Angiotensin Type 1 Receptors
AVV	Adeno-Associated Viruses
C3G	Cyanidin-3-O-Glucoside
CKD	Chronic Kidney Disease
COPD	Chronic Obstructive Pulmonary Disease
COX-2	Cyclooxygenase-2
CRP	C-Reactive Protein
CRS	Cardiorenal Syndrome
CTGF	Connective Tissue Growth Factor
CVD	Cardiovascular Disease
DASH	Dietary Approaches to Stop Hypertension
DHA	Docosahexaenoic Acid
DM	Diabetes Mellitus
DOAJ	Directory of Open Access Journals
eNOS	Endothelial Nitric Oxide Synthase
EPA	Eicosapentaenoic Acid
FGF	Fibroblast Growth Factor
HLA-DR	Human Leukocyte Antigen—DR Isotype
HTN	Hypertension
IL-6	Interleukin-6
LD	Linear Dichroism
MDPI	Multidisciplinary Digital Publishing Institute
MIS	Malnutrition Inflammation Score
MNT	Medical Nutrition Therapy
NF-κB	Nuclear Factor Kappa B
NO	Nitric Oxide
OXPHOS	Oxidative Phosphorylation
PEW	Protein–Energy Wasting
PUFAs	Polyunsaturated Fatty Acids
RAAS	Renin–Angiotensin–Aldosterone System
ROS	Reactive Oxygen Species
S100A1	S100 Calcium-Binding Protein A1
SERCA2a	Sarcoplasmic/Endoplasmic Reticulum Calcium ATPase 2a
SGA	Subjective Global Assessment
SGLT2i	Sodium–Glucose Co-Transporter 2 Inhibitor
SOD	Superoxide Dismutase
SPMs	Specialized Pro-Resolving Lipid Mediators
TCA	Tricarboxylic Acid
TGF-β	Transforming Growth Factor-Beta
TLA	Three Letter Acronym
TNF-α	Tumor Necrosis Factor-Alpha
VEGF	Vascular Endothelial Growth Factor
